# Lecture on Frequent Micturation

**Published:** 1858

**Authors:** G. Owen Rees

**Affiliations:** Physician to, and Lecturer on the Practice of Medicine at Guy’s Hospital


### Lecture on Frequent Micturition.

*Croonian Lecture, delivered before the Royal College of Physicians,* 1827. By G. Owen Rees, M. D., F. R. S., Physician to, and Lecturer on the Practice of Medicine at Guy's Hospital.

Mr. President,—In teaching our art, the plan usually adopted by professors consists in describing each disease in full: symptoms are collected, post-mortem appearances detailed, and the appropriate treatment and diagnosis dilated upon.

This method is valuable in affording us the means of comparing any particular case with the type of the class to which it may belong; imparting a kind of knowledge which all must acquire who are desirous of becoming good diagnostic physicians. There is another method of instruction, however, which may be most usefully combined with that just described.

It consists in selecting for consideration some symptom which experience teaches us to have an important meaning, and to trace it up to each of the causes to which it may possibly be due. At the bedside we meet with symptoms not only of varying character, but of varying value; and the more important are sometimes the least regarded by the uninitiated, inasmuch as they may not be amongst the more painful.

There are some symptoms which, if they be properly studied, restrict the inquiry within narrow limits, while others bear so general a relation to disease that the mind fails to accomplish the analysis which it easily makes where the less general question is involved. It may be asked, how can we acquire this valuable quality of fixing on these more important symptoms—these indications which direct us more immediately to the truth? This is to be attained by experience only, and it is by its possession that some practitioners are enabled (unconscious of the intermediate mental processes) to detect apparently at a glance that which others may have sought for in vain. The physician's diagnosis is the result of a study of symptoms; one by one he values them, and compares them with some set or class of symptoms which he knows constitutes an especial form of disease.—Some inconsistency, perhaps, arises while comparing the symptom he has first selected; in a moment, the train of thought changes. The question, then, arises whether it be consistent in its adjoined phenomena with some other class. The comparison is made again; and eventually an accordance is established leading to the detection of the true nature of the case. Rapidly as this must take place in some minds, still it must needs occur; and this reasoning back from symptoms is what the experts are constantly practising. This method I shall now adopt as the most natural in treating of disease before an auditory already well versed in its general history; and I have selected for consideration, on the present occasion, the symptoms of frequent micturition. This indication, which, under the name of "irritable bladder," is not always so carefully considered as it should be, is productive of extreme misery when present in a marked degree; but there are minor degrees which do not greatly interfere with comfort. The accession of the symptoms may be gradual, and the habits of life of the patient not such as to be materially interfered with by frequent calls to pass urine. On the other hand where social habits or occupation make it desirable that the urine should be held for a few hours, the symptom assumes considerable importance to the patient, however trivial the cause may happen to be, or however easy the cure. According as distress may be produced or not, so we may become acquainted with the symptom, either as the prominent complaint of the patient, or as an incidental circumstance to which he attaches but little importance. Owing to the latter condition, it very frequently happens that this indication is overlooked by the practitioner, so that an obscurity hangs about the case which would at once have been dissipated had the symptom caused more suffering.

I must premise that, in treating this subject, it is not my intention to enter upon the various diseased states of the prostate gland, which we know may produce irritable bladder. This part of the subject is rather in the province of the surgeon than the physician; and digital examination, in many cases will suffice to determine the diagnosis. As compared with the bladder and the kidney, however, the prostate is far from a common cause of the symtom; and the same may be said of the uterus, which, under certain condition of misplacement, produces great bladder irritation.

Frequent micturition, while it may indicate severe inflammatory mischief in the bladder, may, on the other hand, be a purely sympathetic affection, and it is in this latter case that most difficulty occurs in tracing the symptom to its cause. The separation of the subject into these two divisions is not difficult, since inflammatory disease shows a condition of urine not observed when the purely sympathetic affection exists. When frequent calls to pass urine is connected with cystitis, by whatever cause produced, the urine contains excess of mucus, and nearly always pus, and this latter exits in the excreted urine, partly transformed into *adhesive or ropy mucus.* Blood may be seen also, under some conditions, coloring the adhesive mass in the chamber vessels. When the frequent micturition is merely sympathetic, and indicative of some diseased state in a distant part, the urine is not of this character. It is often clear, or merely deposits the urates, and if any other sediment occur, it is not ropy mucus. The long-continued irritation may, however, eventually involve the bladder; then, of course, the first described set of appearances will be observed. We will assume that we have a case showing the symptom of frequent micturition, and on examining the urine, we find ropy mucus as a deposit. This is occasionally colored by blood. Albumen is also dissolved in the urine, owing to the presence of pus.

When the condition has been recognized, we may conclude that the bladder is inflamed, and that one of the three following causes is in action:—

1. Calculus in the bladder.
2. Gonorrhœal inflammation of the bladder.
3. Partial paralysis of the bladder.

The symptoms connected with the first-named cause are so familiar to the profession, that they need not be enumerated here. The points of difference between them, and the symptoms characterizing the two other states are obvious enough when calculus in the bladder produces its full effect. Fortunately, however, for suffering humanity, this is not always so, and then the practitioner may find difficulty in forming a diagnosis. Exploration by sounding by no means sets the question at rest if a negative result be obtained. Calculi, as we all know, may escape detection, even when the most skilful and practised hands hold the sound, and yet at no very remote date from such an exploration, may be easily demonstrated, both to the touch and to the ear, by any one who may have the opportunity of making an examination.

A calculus in the bladder sometimes produces scarcely any symptom whatever, save frequent micturition, with occasional pain at the end of the penis. Again, sometimes, even these symptoms exist but in slight degree, and we have hæmaturia complained of, with scarcely any other indication, the patient being able to bear a good deal of jolting without such pain as usually attends vesical calculus. This is especially observed with mulberry or oxalate of lime concretions. But the state to which I would especially draw attention is that in which the calculus causes hardly any symptoms. Such instances are rare, but we now and then hear persons complaining of frequent call to pass urine, and little else; and on examining the urine the indications of cystitis are sufficiently obvious. The frequent micturition is evidently, then, caused by inflammation of the bladder, but on what this depends is by no means so easily determined. If the patient be sounded, a calculus may perhaps be detected, but if it be not found other causes for the cystisis are sought for, and of course sought for in vain. It becomes of the greatest importance, then, to acquire the power of discriminating in these cases. May it not still be a case of calculus, notwithstanding that no calculus has been found? Much depends on our answering this question correctly. In order to do so we must look to the history of our case, and if we can exclude the two other causes for cystisis—viz., the remains of gonorrhœal inflammation, and the presence of irritating urine, owing to the existence of partial paralysis, we may feel great confidence in declaring it probable that calculus exists, and that the exploration by sounding has not done all it may hereafter accomplish.

Of the two other states above alluded to as capable of producing like symptoms, that of gonorrhœal infection is generally easily ascertained by inquiry into the more early history of the case. I am not here speaking of gonorrhœa with discharge, spreading inflammation to the neck of the bladder, and causing acute suffering. This state of matters cannot well be mistaken; but I allude to cases which arise after many weeks' cessation of gonorrhœa, when, with little or no discharge, gonorrhœal inflammation attacks the bladder. The history here might tell us all, but it so happens that the history is not always forthcoming, and the indications may be, and often are, regarded with anxiety, as possibly connected with a calculus tendency, frequent micturition and ropy mucus in the urine being the prominent symptoms.

A case of this kind occurred to me not long ago. A gentleman who had been the subject of gonorrhœa, and who had recovered from all the first effects of the complaint got married. Shortly after he became the subject of irritable bladder; ropy mucus appeared in the urine, occasionally tinged with blood, and this with a slight loin pain, was all we had to guide our diagnosis. The history, however, sufficed to place the disease before us in the true light.

I shall now notice the third condition giving rise to frequent micturition, and to urine impregnated with the results of inflammation—viz., partial paralysis of the bladder. This is a state which very often commences insidiously. The patient does not feel that he has but partially evacuated his urine, and it is only when the bladder becomes inflamed, owing to the irritation produced by retained and stale urine, that frequent micturition causes him to feel anxiety. He now, perhaps, examines, and finds his urine is passed in an opaque, instead of a transparent state, and that a layer of mucus settles in the chamber vessel. It constantly happens that these cases are not diagnosed correctly for some little time, and instruments may be passed in the belief that the cystitis arises from calculus.

Here we depend almost entirely upon history, and on inquiry we may learn that before the appearance of symptoms our patient had been obliged to hold his urine for a great length of time. He may not have observed after this that he passed but little urine when he had an opportunity of emptying the bladder, nor may he have connected any symptom whatever with the above condition. Sometimes the history is more suggestive.—Complete retention may have existed at some remote date, owing to a distended state of the bladder. An instrument may have been passed, and the urine drawn off, and the patient may not have suffered any symptoms for many months. Then the complaints arise which we are now considering. If the case be neglected, another inconvenience occurs which should at once determine us. This consists in the urine dribbling away in small quantities, but yet incessantly.

Frequent micturition and the urine of inflamed bladder, if taken in connection with the above history, will serve at once to distinguish these cases both from calculus and from gonorrhœal cystitis. They are often at first mistaken for the former, and nearly all the patients I have seen with this affection have had the sound passed, and sometimes by more hands than one, without any light being thrown upon the subject.

Before proceeding to the second division of my subject, I would say a few words respecting two forms of cystitis noticed by authors. First, as to cystitis occurring more or less in the character of an idiopathic affection, as caused in irritable constitutions by exposure to cold. This disease has been described, I believe, only because some mechanical or chemical cause has been overlooked. Secondly, we hear of a gouty cystitis. This, which has been described as an immediate consequence of the gouty diathesis, may, I believe, be more correctly regarded as secondarily produced by calculus affection. The irritable bladder in some gouty persons may be clearly traced to sympathetic irritation produced by renal calculus, and cystitis may eventually occur; but I am by no means inclined to believe the bladder liable to specific gouty inflammation. One argument which has been used in favor of the specific nature of this imflammatory state, is founded on the fact that relief has been obtained by the administration of colchicum; and were this drug quite inefficacious in all other inflammations, and invariably successful in relieving gout, some weight might be attached to the argument; but in the present state of therapeutical science it bears but little on the question.

Before considering the causes of *sympathetic* irritation producing frequent micturition, I must refer to a question which may very possibly suggest itself to some of my hearers. It may be thought that though sympathetic affections may require analysis with regard to the symptoms under consideration, frequent micturition must be expected in every disease connected with an inflammatory state of the urinary apparatus: in point of fact that it is a necessary concomitant. This, however, is by no means the case; and there is one cause for this form of urine indicative of inflammation requiring more especial notice, as not the slightest irritability of bladder need exist, though the urinary symptoms are otherwise closely allied to those just described. This happens when a calculus becomes fixed in the ureter. Under these circumstances there may be merely such sensations about the urethra as are easily accounted for by the altered nature of the urine, which is generally highly alkaline, and deposits the earthy phosphates if kept. We are well aware how irritating the presence of calculous matter is in the kidney, and in the bladder; and so long as the inflammatory state of the urine is observed we always expect frequent micturition. In the case I now refer to, however, the calculus gives little inconvenience. The history of its first leaving the kidney may be remote, but inquiry will lead to the point, and severe pain be described as having occurred at some date antecedent to the appearance of mucus and pus in the urine. I doubt not that some of my hearers may have seen a case or more in which post-mortem-examination has shown the ureter of one side blocked up by calculus, the ureter above greatly distended, and the organ probably undergoing gradual destruction.

Having now spoken of cases of frequent micturition in which the urine gives indications of cystitis, I will proceed to consider the other division of the subject, including those cases in which the secretion is not so materially affected, and which arises from sympathetic irritation of the bladder. This class is a somewhat numerous one. One of the most common causes for the symptom exists in the presence of kidney disease, and especially that known as morbus Brightii. Here the frequent micturition for the most part occurs at night, and the patient may disregard the inconvenience for a length of time. Scarcely any other symptom of the disease may be present, and if this one be not sufficiently valued by the practitioner, the malady may escape detection altogether. There may be slight dyspepsia, and perhaps a somewhat anæmiated look. The urine may be clear, and no deposit indicating cystitis present; but the bladder is irritable. Under these circumstances, the question as to the possible albuminous condition of the urine should at once suggest itself. If this be detected, then it becomes necessary to deal with the question of albuminous urine in its relations to disease, and to determine whether we may refer it in this particular instance to granular degeneration of the kidney. What is it necessary to do in order to effect this? I need not remind my hearers that albumen may be present in the urine merely as a concomitant with pus or with blood. The first step, therefore, should be to ascertain whether or not either or both these be present, and microscopic examination is rarely necessary in order to effect this, as far more than a microscopic quantity of the corpuscles of these fluids must be mingled with the urine to render it albuminous in any marked degree. We will assume that these sources of fallacy are removed, that the urine contains the serous part only of the blood, as in morbus Brightii, and now the question may be asked, upon what other states may this depend? This is an old query. It was the difficulty which occurred to the minds of practitioners when Dr. Bright's discoveries were promulgated. It was thought unlikely that albuminous urine should possess such especial and exclusive significance. Many assertions were made tending to lessen the value of the indication; and had there been any amount of truth in the allegations, albuminuria would long since have been recognised as a very common symptom, existing in numerous diseases, and even occurring under conditions scarcely to be distinguished from perfect health. Thus it has been said that eating pastry or drinking milk in quantity will cause albumen to appear in the urine, and a mercurial course has been supposed to produce the same effect.—Some believe it a common concomitant of ordinary colds, and as easily produced by any interference with the function of the skin. The attention I have paid to the subject, with ample opportunities for experience, enables me very confidently to contradict the above statements; but it may not be out of place to mention one or two other objections to the exclusive nature of this indication, possessing more claims to notice.

Albumen appears occasionally in the urine during gestation. Some women have been known to excrete it during every pregnancy, and no evidence has subsequently appeared to prove the existence of diseased kidney. This fact was first noticed by Dr. Lever. Albumen, again, may nearly always be detected in the urine of women suffering from puerperal convulsions. Again, during the progress of cholera asphyxia, the urine frequently becomes albuminous. It is said to assume this state also in typhus; but if this ever occur it is a rare concomitant of that fever, and if it be present it will be well to look to the kidney.

The conditions just described are obviously such as but little interfere with the diagnostic value of albuminous urine, inasmuch as they are easily recognized.

I would, lastly, notice those statements which, were they proved by the facts adduced, would entirely destroy the value of albuminous urine as a guide to diagnosis. A writer of considerable chemical acumen, but evidently ill-informed in the phenomena of disease, and who, like many other chemists who have meddled with pathology, has done much to confuse a very important subject, has declared that "albuminous urine has now been so frequently observed in numerous diseased states of the organism, independent of Bright's disease, that the idea has long been abandoned that granular degeneration of the kidneys always occurs where we have albuminous urine." So far so good. If we except the word numerous, what I have already said is quite in accordance with the view propounded; but our author goes on to say, that albumen exists in the urine of blooming health, and without giving us the sequel, contents himself with describing albuminuria and robustness. The case (if correctly reported) is that of a confirmed, but perhaps early, stage of morbus Brightii. While suffering from a mild catarrho-rheumatic affection, the author found a trace of albumen in his own urine.—Next we have a case quoted in which the presence of albumen may have been simulated with the phosphates; but even if we allow that albumen was present, there is no account of the after history, nor of the post-mortem, to set the question at rest as to the existence of morbus Brightii. The patient, in this last case, was the subject of pneumonia, a disease which often complicates chronic albuminous nephritis, and in all probability, if albumen really existed in the urine, this patient had a degenerated kidney.

We next have two cases which are almost certainly true morbus Brightii; the one regarded by the author as rheumatism, the other as dropsy with albuminous urine, but *not* with kidney disease, because as he writes, "*the patient complained of no pain* (*even on pressure*) *in the lumbar region.*

These statements, which are to be found in the writings of the late Professor Simon, of Berlin, have done much to interfere with progress. The merest tyro of our schools could have told our author that patients with morbus Brightii scarcely ever complain of lumbar pain—that it is by no means to be expected, even in the severest cases—that its presence is the exception, its absence the rule; and that the same remark applies to pain produced *by pressure* over the region of the kidneys.

The carefully collected records of hospitals have now satisfactorily determined this question; and it may be confidently stated, that albuminous urine indicates either a degenerated kidney, or some state of the organ preceding degeneration; and that the sources of fallacy are not material, consisting as they do of conditions which can be easily recognized—such as pregnancy, puerperal convulsions, and cholera asphyxia. I would add a precaution here, however—viz., not to draw any inference from the examination of urine obtained after death, when albumen may be often detected as a result of transudation.

Among the causes for frequent micturition (irritable bladder) we find brain affection has been enumerated; but from the accounts we read of these cases it appears highly probable the cerebral condition described was merely one of the concomitants of kidney disease, which it was not possible to recognise before medical literature had been enriched by the writings of Dr. Bright, and before his discoveries had been promulgated. Surgical writers have spoken of this form of irritability.

There is a state of urine to which I would now direct attention, as occasionally productive of frequent micturition but which easily admits of relief. It consists in an increased acidity generally observed in gouty subjects. Uric acid is occasionally seen as a deposit, but not always so at the commencement of the malady. These cases sometimes occur in connexion with albuminuria, and the fact is an important one, because it seems to constitute a point of difference in the views of those whose acquaintance with the subject of albuminuria is profound, and whose experience has been most extended. The question lies thus:—If a patient passing uric acid pass also albuminous urine, and if these conditions continue many months, is the case necessarily one of kidney degeneration? M. Rayer, with whom I had a most interesting consultation last summer, holds that the gouty or uric acid diathesis may affect the kidney, causing a degeneration of its structure (Bright's disease—"nephrite chronique albumineuse" of the French,) or it may, on the other hand, cause a discharge of albuminous urine (in consequence of the uric acid crystals irritating the tubules,) without any degeneration of the kidney occurring as a consequence. This latter state may last, it is said, for months, and, according to this doctrine, even after some year or two, the case is not to be condemned as necessarily connected with degenerate kidney. The concurrence of uric acid deposit with albuminous urine was noticed by Dr. Prout, who even went so far as to believe in a necessary connection between the two. Uric acid, however, is so familiar to us as a deposit without albuminous urine that this necessary relation cannot possibly exist. When, however, the two happen to occur together, we have a condition admitting of the two interpretations just given.

The persistence of albumen month after month has been considered by Dr. Bright and his followers as necessarily indicative of organic change in the kidney, and that this is generally the case cannot be denied. We see the fact constantly proved in our hospitals; but the question still lies open as to whether the uric acid crystals may not cause albuminous urine to appear for many months, or even longer, by irritating the tubules. We frequently hear of cases of albuminous urine going on for years and years without any very serious inconvenience to the patient. We hear also of cases which have been cured, the patient remaining well for years. Let us consider whether we are to believe in this less hurtful albuminuria, and whether the cases which appear to admit of relief are those in which the irritation of uric acid crystals is causing the albuminous discharge, without any organic disease being necessarily present in the kidney. Since my attention has been directed to the point, I have had two opportunities of observing albuminuria in connection with the uric diathesis. The albumen was present in abundance, but disappeared under alkaline treatment in one case almost immediately.

Here we may have had an early case of Bright's disease in a gouty subject, which was relieved by treatment, or, it may have been a case of irritation of the tubules by uric acid, as described by M. Rayer. For my own part, the rapid relief from alkaline treatment inclines my belief strongly to the latter view, and it appears probable, that though gouty subjects are prone to Bright's disease, they yet may pass albuminous urine from another cause.

But what are we to say to cases in which the albuminuria persists for months or years? Are we to believe in the possibility of this discharge of albumen continuing, without the kidney being organically diseased? My conviction was strongly against such a belief, but that opinion has been somewhat shaken of late.

It has always been a puzzle to explain the old case referred to, in which morbus Brightii is born with apparent immunity, and I am by no means satisfied but that an explanation may be found for this apparent anomaly in the condition noticed by M. Rayer.

I have at present a patient under care, who has passed albuminous urine for six years and more, whose state of health, so far as we can judge, is perfect. Strong, active, and energetic, she repudiates the idea that she is an invalid. She is of a gouty family, and has occasionally passed uric acid. The urine has never been of low specific gravity, no fibrinous casts have ever been detected, and there is no evidence whatever that the blood or other fluids have become degenerated in any way. Alkaline treatment has answered well in this case. Under its use, the albumen has disappeared for days and days together, and though it is still occasionally found, it is always in very small quantity.

If ever a case existed capable of clearing up the difficulty I have alluded to, this is the one. Can the gouty diathesis, with its uric acid crystals, be causing the albuminous urine, or have we a case of true morbus Brightii? Post mortem examination alone can determine this.

The next cause for frequent micturition which I shall notice consists in the presence of calculus in the kidney. Here the irritation is often most excessive, and there is the greatest difficulty in persuading the patient that his bladder is not diseased. These cases are generally amongst the most satisfactory that can fall under our care. Little is to be done, and doing little or nothing is often a hazardous step as regards the fame of the practitioner. You cannot expect the suffering and ignorant patient to believe in your declaration that he must still suffer on till some lucky accident either expel the calculus or enable it to become encysted, and he will fly to those who in accordance with his own views, may proceed to treat him for disease of the bladder. I have known these cases regarded as dependent on irritation of the neck of the bladder caused by stricture, and have seen the most lamentable results brought about by the violent measures resorted to for relief.

The correct diagnosis is not very difficult. The history generally tells of hæmaturia, probably at some remote date, and of occasional loin pains and uneasiness. The general health at first is but little disturbed. The urine shows none of the indications of cystitis, is generally clear at first, but in old cases it becomes slightly clouded. This cloudiness is dependent on the presence of pus, which exists in small quantity. We must not expect to find hæmaturia a warning symptom. In many instances it is certainly present, and our diagnosis is then more easily made, but if hæmaturia do not exist at the time of our seeing the patient, and when we have an imperfect history to guide us, the case cannot be determined so easily.

Frequent micturition, with small quantities of pus in the urine, loin pain, and lassitude, if we have an early history of hæmaturia, should guide us to diagnose renal calculus; and even if frequent micturition and a small quantity of pus be the only symptoms, we shall generally be right in giving the above opinion, even if history fail to afford us evidence of hæmaturia.—The presence of a small quantity of pus in the urine would appear easily explained in its relations to renal calculus.

The hollowing out of the nephritic structure, which we find occurring in order to make room for calculi about to become encysted in kidneys, must have been effected by a gradual process of disintegration, and this we know is preceded by inflammation. The purulent discharge would thus seem to attend the formation of a convenient cavity for the lodgement of the calculus. So long as this action is going on, the patient will pass pus in the urine, and it may be some years before matters are adjusted. The constitution has much to go through. A scrofulous taint leads to abscess in the kidney and death. The more fortunately constituted generally do well, provided they can be induced to avoid the catheter and the sound.

In speaking of the condition of the urine in this calculous affection of the kidney, I have made use of a somewhat indefinite expression—viz., "a small quantity of pus." By this I would wish my readers to understand an urine depositing a yellowish-white sediment, but not in such quantity that the patient's attention need be attracted by it. It renders the urine but slightly turbid as it is passed, or when the deposit is shaken up in it.

This is the general state of things when nephritic calculus is encysting, or when it fails to find its way down the ureter, provided constitutional causes do not interfere to produce suppurative disease which may appear in the form of pyelitis or of general abscess of the kidney. This purulent impregnation is constant, and if it fail to show itself, so as to be evident to the unassisted eye, the microscope rarely fails to demonstrate the presence of pus so long as the bladder is irritable.

There is a cause for frequent micturition so nearly connected in its symptoms with that last noticed, that it naturally suggests itself in this place. It consists in a state of kidney known as strumous kidney, or phthisical kidney, as some authors have designated it. If calculous disease develop itself in a strumous subject, we find very early that abscess results, but in all subjects some amount of pus may be expected during the time of encysting. In phthisis of the kidney, however, the bladder becomes irritable, without any evidence of a calculous disposition; and we find that pus can be clearly proved in the urine. The symptoms are generally at first considered to depend on calculus, and it too often happens that the disease has made great progress before the real state of the case becomes evident. The symptoms are at first nearly identical with those of nephritic calculus. The same degree of sharp lumbar pain, however, is not present, and there is no history of hæmaturia; but the symptoms presenting themselves at the time of examination bear a striking similarity; and if the previous history be not ascertained, a diagnosis is next to impossible. It is both for the advantage of the practitioner and of the patient that this distinction should be early made; for if calculus be the exciting cause, of course our prognosis will be more favorable.

The two points for consideration are—1st. The diathesis of the patient. 2nd. The history as to hæmaturia. If frequent micturition and purulent urine, such as I have described, be present in a strumous person, and we have no history of hæmaturia, we may diagnose phthisis of the kidney. If frequent micturition and purulent urine co-exist with a history of hæmaturia, then, in all probability, there is calculus. We must not conclude, however, that because calculus is present we have no fear of the worst results, for if the patient be of strumous habit, abscess may result as a consequence. In all cases, however, the history of hæmaturia is an advantage, inasmuch as even should the patient be strumous, the calculus may be voided, and the exciting cause of mischief being thus removed, the kidney may recover itself, and the patient do well.

It is not many months ago that I saw a remarkable strong young man suffering from loin pains and general malaise, in whose urine small quantities of pus were nearly always present. The case interested me much, and I looked with some anxiety for the previous history. There was a strumous diathesis; and from the moment I made my examination, I felt certain that all depended on the history involving hæmaturia or not. In any case, the strumous diathesis made it a serious affair; but in the absence of hæmaturia, the only conclusion which could be arrived at was that the nephritic mischief had resulted purely from struma. As phthisis of the kidney progresses, we may have enormous quantities of pus evacuated. It is only therefore, to the commencement of the disease that my remarks apply; when, with frequent micturition, we have the slightly purulent secretion simulating calculous mischief.—*London Lancet.*

